# Effects of Itxasol© Components on Gene Expression in Bacteria Related to Infections of the Urinary Tract and to the Inflammation Process

**DOI:** 10.3390/ijms222312655

**Published:** 2021-11-23

**Authors:** José M. Cela-López, Claudio J. Camacho Roldán, Gorka Gómez-Lizarraga, Vicente Martínez

**Affiliations:** 1 Celavista, Parque Científico de la UPV/EHU, Edificio Sede, Barrio Sarriena, s/n, 48940 Leioa, Bizkaia, Spain; Jose.cela@naturemimetix.com (J.M.C.-L.); claudio.camacho@naturemimetix.com (C.J.C.R.); gorka.gomez@naturemimetix.com (G.G.-L.); 2 Naturemimetix, Parque Científico de la UPV/EHU, Edificio Sede, Barrio Sarriena, s/n, 48940 Leioa, Bizkaia, Spain

**Keywords:** Itxasol©, β-arbutin, biofilm, umbeliferone, N-acetyl cysteine

## Abstract

Urinary tract infections (UTIs) represent a health problem of the first magnitude since they affect large segments of the population, cause increased mortality and comorbidity, and have a high incidence of relapse. Therefore, UTIs cause a major socioeconomic concern. Current antibiotic treatments have various limitations such as the appearance of resistance to antibiotics, nephrotoxicity, and side effects such as gastrointestinal problems including microbiota alterations that contribute to increasing antibiotic resistance. In this context, Itxasol© has emerged, approved as an adjuvant for the treatment of UTIs. Designed with biomimetic principles, it is composed of arbutin, umbelliferon, and N-acetyl cysteine. In this work, we review the activities of these three compounds concerning the changes they produce in the expression of bacterial genes and those related to inflammation as well as assess how they are capable of affecting the DNA of bacteria and fungi.

## 1. Introduction

There are around 150 million urinary tract infections (UTIs) annually with a very high associated socioeconomic cost (in the USA alone, this is estimated to be in the order of $3.5 billion [1,2]). These infections affect women more than men, a fact that is related to the shorter length of the urethra in women than in men. Bacteria are capable of colonizing the urinary tract and moving up through it to reach the kidneys [3]. The pathology they can cause ranges from asymptomatic processes, to cystitis, and can become complicated even leading to cases of pyelonephritis [4,5]. As mentioned, these infections are more frequent in women and are associated with low socioeconomic levels related to poor hygiene in the menstrual period [6], sexual intercourse-postcoital UTIs [7], use of barrier contraceptive methods [8], and an increase with age related to alteration of hormone levels [9]. In the case of men, these infections are recurrent and are associated with cases of prostatitis, obstruction in the urethra, and benign hyperplasia [10]. Regardless of gender, the use of catheters is closely linked to the appearance of UTIs [11]. Reinfections in the case of UTIs are quite common although they vary according to age, with common occurrence of relapse after the first diagnosis [12].

One of the major problems associated with the use of antibiotics for the treatment of UTIs is the appearance of resistance to antibiotics that causes an increase in mortality, morbidity, and high socioeconomic costs. In addition to resistance to antibiotics, there are also collateral effects such as kidney damage, changes and alterations to the intestinal flora that cause digestive problems, and alterations to the metabolism and immunity [13,14,15,16].

The main bacteria associated with UTIs are *Escherichia coli* and *Pseudomonas aueroginosa* [17]. In the case of infections produced in hospitals, it has been shown that in addition to *Escherichia coli* and various enterobacteria, in this context, other bacteria such as *Klebsiella* spp. and *Enterococcus* spp. have been isolated from patients [18].

On the other hand, the formation of a biofilm by different bacterial strains has been related to an increase in resistance to antibiotics, as well as an increase in mortality and morbidity of diseases associated with infections caused by them [19,20]. The biofilm is an extracellular structure that is made up of sugars, lipid proteins, and molecules derived from DNA, which helps to spread bacteria and make them more resistant to antibiotics [21].

The most common antibiotic treatments for the management of UTIs are fosfomycin, nitrofurantoin, and quinolones to treat cystitis, and in the case of pyelonephritis, third-generation cephalosporins. In addition, in the case of administration for prophylactic purposes, trimethoprim + sulfamethoxazole, nitrofurantoin, cephalexin, and fosfomycin are used, while in the case of complicated cystitis, ciprofloxacin, levofloxacin, cefpodoxime, and ceftibuten are administered. In the case of having to administer a second line of treatments for pyelonephritis, cefepime, piperacillin/tazobactam, gentamycin, and amikacin are administered [22]. Table 1 describes the main antibiotics used against UTIs and their mechanisms of action.

Among the different causes generating resistance are their overuse, which enhances a favorable selective pressure when resistant strains spread and the ease with which resistance factors to treatments are transmitted between them. In addition, another aspect to take into account is bacterial resistance, understood as the ability of the system to return to the initial conditions after having suffered a disturbance to its state; in the case of bacteria, this implies temporary resistance to antibiotics that establishes a series of bacterial subpopulations. This concept is linked to genetic and non-genetic aspects that are manifested in three concepts: tolerance, resistance, and hetero-tolerance [28].

In particular, bacterial resistance can be associated, in the case of pathogens that cause UTIs, with the formation of biofilms. Biofilms are heterogeneous structures composed of bacteria and surrounded by a matrix [28]. These biofilms are related to the development of resistance to antibiotics, and it has been shown that the bacteria that produce these structures have a resistance to antibiotics that is 100 to 1000 times higher than that of bacteria that do not generate them.

Other causes of the appearance of resistance are to do with the characteristics of the host. It has been shown in an animal model that reinfections of UTIs are capable of altering the host cells, causing changes at the transcriptional level that affect the maturation of epithelial cells and that can remodel the composition of the epithelium, together with changes in inflammatory processes dependent on cyclooxygenase 2 [24].

The fact that certain pathogens can relatively easily infect the urinary tract is often related to their ability to adhere to the urinary epithelium through the presence of fimbriae or pilum, or to the capability of bacteria to adhere to each other by expressing adhesins, form biofilms, or generate molecules that can mask the natural response to lipopolysaccharide (LPS) [29,30]. Thus, the expression of certain genes by bacteria related to the production of fimbriae, biofilms, toxins, and adherence factors is related to the appearance of UTIs and to recurrent infections [31,32,33,34]. In addition, part of the damage produced by these bacteria is related to the inflammation processes caused in these infections, as in the case of pyelonephritis, a major complication of UTIs [35,36]. As we will demonstrate in this article, the mechanisms of action of the components of Itxasol© are directly related to these two important aspects: modulation of the expression of virulence genes, and regulation of inflammation.

Our objective with this work was to compile published evidence to relate each of the components of Itxasol© with modifications in the expression levels of genes in different cell lines and animal models related to the inflammation process, and changes in the bacterial genomics of pathogens related to UTIs.

## 2. Itxasol© and Its Formulation

Given the high recurrence of UTIs and problems associated with the use of antibiotics, it is necessary to explore new tools to combat the pathogens that cause these infections. In this context, the recently approved Itxasol© emerges as a new therapeutic option for UTIs′ treatment. Itxasol© has been designed following biomimetic principles and it is an effective compound against these infections that do not present side effects and do not generate microbial resistance. Biomimetics is defined as a science that studies nature as a source of inspiration for innovative technologies to solve human problems, through models of systems (mechanics) or processes (chemistry), or elements that imitate or are inspired by nature [37]. Itxasol© has been recently authorized as a food supplement with authorization number C.N. 203621.5 in Spain and can be used alone or in combination with the current antibiotics to treat complicated UTIs.

**Figure 1:**
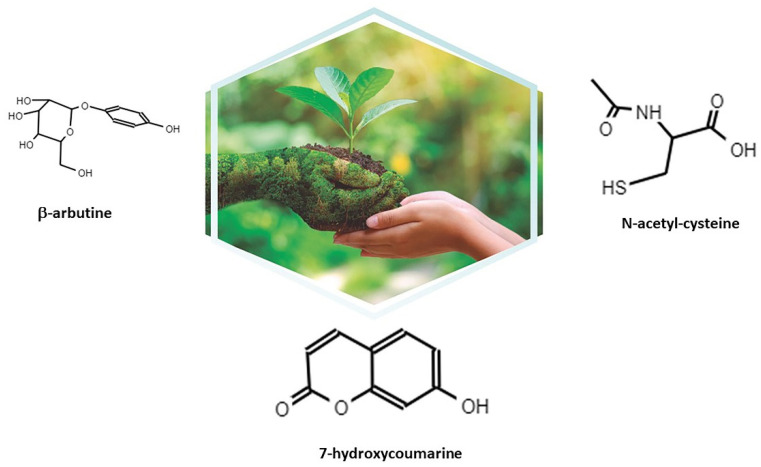
Molecules that form Itxasol©: β-arbutin, umbelliferon (7-hydroxycoumarine), and N-acetyl cysteine (NAC).

Itxasol© is composed of three molecules: β-Arbutin, umbelliferon (Umb), and N-acetyl cysteine (NAC) (Figure 1). Itxasol© is orally administered in capsules each containing 300 mg of Umb, 150 mg of β-arbutin, and 150 mg of NAC. Umbelliferon (or 7-hodroxi-2h-1-benzopiran-2-ona or 7-hidroxicumarina) comes from extracts plants of the Apiaceae family (umbeliferas), such as carrot, coriander, and garden angelica, and the family Asteraceae or the hydrangea leaf. It is also found in the *Justicia pectoralis* (acathaceae) plant. We obtained it from *Artemisia capillaris*. β-Arbutin (or hydroquinone O-beta-D-glucopyranoside) comes from extracts of *Ursi uva* (gayuba). N-acetyl-L-cysteine is obtained by chemical synthesis. The most important aspect of the Itxasol© formulation is its multimodal action mechanism. It is not only a bactericidal and bacteriostatic antibiotic, but also an antibiofilm, anti-inflammatory, antioxidant, and nephroprotective protector of the microbiota and the environment. Of note, in its use, no antibiotic residues are delivered to the environment. Other molecules such as D-mannose and curcumin from cranberry extracts have been proposed as alternatives for the treatment of UTIs. Both molecules present as a mechanism of action to prevent the adhesion of bacteria to the urinary epithelium. However, these molecules lack antibacterial activity, their dose of use is not well established, and in the case of D-mannose, it presents side effects such as diarrhea [38,39,40]. Here, we will briefly review the characteristics of each of the molecules that form Itxasol© related to their action against UTIs.

### 2.1. β-Arbutine

Arbutin is a glycoside that has been used in different areas of Europe, America, and Asia as a traditional treatment for urinary infections and that is extracted from the genera *Bergenia*, *Ainsliaea,* and *Calluna*. This molecule, once metabolized by the body, becomes hydroquinone; one of its greatest advantages is that approximately 65% of the arbutin is converted into HQ. The mechanism of action of HQ is largely related to destroying the bacterial wall and causing the death of the bacteria [41,42]. The antimicrobial activity of arbutin has been tested in different bacteria, both Gram-positive and -negative, and even fungi. Bacteria and fungi that have been shown to be affected by arbutin′s action are *Bacillus subtilis, Candida albicans*, *Enterococcus faecalis*, *Escherichia coli*, *Pseudomonas aeruginosa*, and *Staphylococcus aureus* [38,41,43,44].

In addition, it has recently been shown that this molecule is capable of impairing the formation of the biofilm produced by *Streptococcus mutans*, thus inhibiting the production of glucosyltransferases, which are responsible for producing the polysaccharides of the bacterial cell wall, which are related to adhesion among the bacteria [43].

### 2.2. Umbelliferon (Umb)

Umbelliferon, or 7-hydroxycoumarin, is a molecule derived from coumarins that is present in different fruits and plants, with protection functions of the pancreas and nervous system. As well as this, it has demonstrated anti-arthritic and antitumor activities [44,45,46,47]. The antibiotic activity of Umb was described in 1978; however, since then, little progress has been made in understanding its mechanism of action [48]. In addition to its antimicrobial activity, umbelliferon has been described as a very potent antifungal agent [46,49]. Some of these actions have been related to the increase of oxidative stress and subsequent apoptosis, as we explain in a further section.

### 2.3. NAC

NAC is a molecule known for being a powerful mucolytic and for regulating cellular oxidative stress levels, which makes it a molecule with a multitude of potential applications [50]. The antibiotic action of NAC is related to different mechanisms and not only those related to its antioxidant capacity but also to its interaction with gene expression and even its ability to degrade DNA. Although its exact mechanism is not known, NAC is capable of destroying biofilms formed by bacteria and fungi. In addition, its role as a renal protector has been demonstrated in a study in a model of renal ischemia in rats, where its administration produced an improvement in the renal biochemical parameters of rats treated with NAC after ischemia establishment [51,52].

## 3. Influence of Itxasol© in the Expression of Genes Related to Inflammation and Changes in Bacterial Genomics

### 3.1. β-Arbutin

Besides the described antimicrobial action, arbutin is also known for its anti-inflammatory action that modulates the genetic expression of different genes involved in this process. Table 2 summarizes the main actions of β-arbutin in this context. 

It has been demonstrated that in microglia cells, the administration of β-arbutin to b2 microglia cells also treated with lipopolysaccharide (LPS) led to a reduction in the expression of inducible NO synthase (iNOS) and the molecule involved in cell adhesion such as ninjurin 1 (Ninj1). In addition to reducing the gene expression of the aforementioned genes, arbutin reduced the production of inflammatory molecules and cytokines such as IL-1β, TNF-α, MCP-1, and IL-6 [53] Besides this, β-arbutin is useful to reduce oxidative stress. Thus, in prostate cancer cell line LnCAP and fibroblasts treated with tert-butyl hydroperoxide to increase their oxidative stress, arbutin was also able to reduce the expression levels of p53 in the fibroblast and increase the BAX/BCL-2 ratio, changes that were related to increased apoptosis in LnCAP cells [54]. The authors of the study concluded that while β-arbutin has a protector action against oxidative stress in fibroblasts, it has a proapoptotic action in a tumoral cell line such as LnCAP.

Apart from inflammatory processes, it has been shown that β-arbutin is capable of promoting the proliferation and differentiation of MC3T3 osteoblasts. In this context, the administration of arbutin promotes an increase in the expression of type I collagen and genes related to differentiation such as one γ-carboxyglutamate protein (BGLAP), Sp7 transcription factor (SP7), and runt-related transcription factor 2 (RUNX2) [55]. In addition, arbutin in combination with rosmarinic acid reduces the expression of osteoclast precursor cells such as RAW 264.7 of the genes related to osteoclast differentiation genes, nuclear factor of activated T cells cytoplasmic 1 (NFACTc1), and the following markers of osteoclast differentiation: matrix metalloproteinase-9, tartrate-resistant acid phosphatase, and cathepsin-K. This reduction resulted in a decrease in the osteoclast activity [56].

One of the concerns when using arbutin is that it can be considered nephrotoxic. It is noteworthy that the antibiotic activity of this molecule respects the integrity of the DNA of epithelial cells in a rat model, and in the case of humans, in isolated peripheral blood lymphocytes [57,58].

### 3.2. Umbelliferon (Umb)

In this section, we describe the main changes related to gene expression that Umb produces in bacteria and inflammation. Table 3 summarizes the major findings.

Umb has been described as capable of preventing the formation of biofilms in strains of *Staphylococcus epidermidis* resistant to methicillin. This action is carried out by reducing the expression of genes that code for factors involved in the formation of the bacterial biofilm, others that favor bacterial invasion such as *agrA*, those related to the production of hydrolases, and those of bacterial adhesion such as *icaD, atlE, aap, bhp, ebh, sdrG,* and *sdr*. Specifically, Umb is capable of reducing the expression of genes encoding exopolysaccharide (PIA) synthesis *icaD*, virulence and biofilm formation (*agrA*), intercellular adhesion and accumulation (*aap* and *bhp*), autolysin/adhesin (*atlE*), and ECM-binding protein (*ebh, sdrG,* and *sdrF*) [59]. It is noteworthy that coumarins have an antifungal activity against *Candida albicans*. This activity is related to the production of apoptotic effects such as the migration of phosphatyl serine to the external face of the plasma membrane, DNA fragmentation, and nuclear condensation [60]. Umb has shown that it is capable of reducing the virulence of the *Escherichia. coli* O157:H7 strain that is the main cause of hemorrhagic colitis. The use of Umb produced a reduction in the expression of genes related to bacterial motility and the creation of the extracellular matrix (Curli genes). This decrease in expression resulted in a reduction in the number of fibers and bacterial motility [61].

**Table 3:** Actions of Umb.

Action/Finding	Reference
Downregulation of genes involved in biofilm production and adhesion	[60]
Downregulation of genes related to production of extracellular matrix and motility	[61]
Attenuation of DNA damage for oxidative stress	[62]
Reduction of inflammasome	[63]
Reduction of apoptosis of kidney cells	[64]
Produces DNA fragmentation in oral carcinoma cells	[65]
Cell cycle arrest in G1 apoptosis of adenocarcinoma cells	[66]

Umb has also demonstrated anti-tumor and anti-arthritic activities due to its antioxidant and anti-inflammatory properties, as shown by multiple studies supporting these characteristics [46,47,64,65]. Related to its anti-inflammatory activity, Umb has shown in an in vivo study with lead-treated rats that it is capable of attenuating the damage to DNA due to oxidative stress and that it is capable of reducing the gene expression of genes such as BAX, while increasing that of BCL-2 [62]. All of this led to a reduction of lead-mediated testicular damage in this animal model. Concerning the beneficial effects of Umb in relation to kidney inflammation produced by antibiotics, it has been shown that Umb is capable of attenuating this inflammation through the inhibition of the TLR-4/NF-κB-p65/NLRP-3 and JAK1/STAT-3 signaling pathways. In particular, Umb is capable of reducing the expression of the ERK1/ERK2, TLR-4, and p38MAP genes, as well as increasing the expression of IkBα, which leads to a reduction of the inflammasome [63]. Similar results were obtained after treatment with Umb in other models of kidney damage, with methotrexate that led to the reduction of apoptosis of kidney cells [64], and in another model of kidney damage with cisplatin, where an increase in the expression of genes such as CREB, SIRT1, FOXO-3, PPAR-γ, and NRF2 was associated with protection against nephrotoxicity [64].

Umb has been shown to be capable of causing oral carcinoma cell death through apoptosis. In experiments carried out in KB cells, it has been shown that the administration of Umb produces an increase in oxidative stress in these cells that induces cycle arrest in cells between G1 and G0, depolarization of the mitochondria, and DNA fragmentation leading to cell apoptosis [65]. In relation to abnormal cell growths, it has also been shown that treatment with Umb produces a reduction in the proliferation of a cell line of benign prostatic hyperplasia. This activity is due to the regulation of the STAT3/E2F1 axis. Furthermore, in an animal model (in rat) of benign prostate hyperplasia, Umb is capable of reducing the size of the prostate, related to reduction of the expression of cell nuclear antigen and p-STAT3 genes [65]. It has also been described how coumarins can be effective against lung carcinoma cells through the induction of apoptosis of these cells. Thus, the actions of coumarin and Umb in non-small lung carcinoma cells produced the arrest of the cell cycle in G1, and with high doses of these compounds, produced apoptosis in adenocarcinoma cells [66].

### 3.3. N-acetyl-L-cysteine (NAC)

NAC can act against pathogens such as *Escherichia coli* and *Enterococcus faecalis*, preventing them from invading epithelial cells of the bladder [67]. This fact has been related to how the administration of NAC to cells can alter the synthesis of membrane proteins related to the recognition of bacteria in their first step, which is that of adhesion [68]. In addition, in other studies, NAC has shown to be capable of degrading various polysaccharides secreted by bacteria that bind to the matrix that forms in biofilms of pathogens such as *S. aureus* MSSA and MRSA, *Pseudomonas aeruginosa*, and *Helicobacter pylori* [69,70,71]. The mechanism of action seems to be linked to the possibility that weak acids can enter the bacteria and directly destroy the DNA of the bacteria [72]. In addition, bacterial biofilms are composed of various molecules such as sugar lipids, protein, and even nucleic acids derived from DNA such as phenazines [73]. The right connection of these molecules is essential for the maintenance of the biofilm. Although there are no studies carried out to date, it is important to know whether NAC is capable of interfering with these actions.

Regarding the actions of NAC concerning inflammation of the urinary tract, it should be noted that NAC has been shown to be effective in LPS-induced bladder fibrosis. In this context, the administration of NAC inhibited an increase in the expression of genes related to fibrotic processes such as Tgfb2, Tgfb3, Smad2, Smad3, Cxcl10, and Card10. This was related to a decrease in hemorrhagic processes and the presence of lymphocytic infiltrates in the renal tissue [74]. NAC has also been shown to be effective as a kidney protector in sepsis. In a rat model, NAC treatment resulted in reduced expression of genes related to inflammation, tumor necrosis factor α, interleukin (IL)-1β, IL-6, and IL-8. In addition, there was a decrease in the expression of genes related to apoptosis such as caspase-3, caspase-9, and cytochrome c, and as a consequence, the number of apoptotic cells in the kidneys of these rats [23]. The actions of NAC in this context are summarized in Table 4.

## 4. Conclusions and Future Directions

Given that UTIs have a high socioeconomic cost for society, there is a need to provide new ways to combat these infections. Itxasol© is a biomimetic compound that has antimicrobial, anti-biofilm, and anti-inflammatory characteristics that are very useful to fight this type of infection.

**Figure 2:**
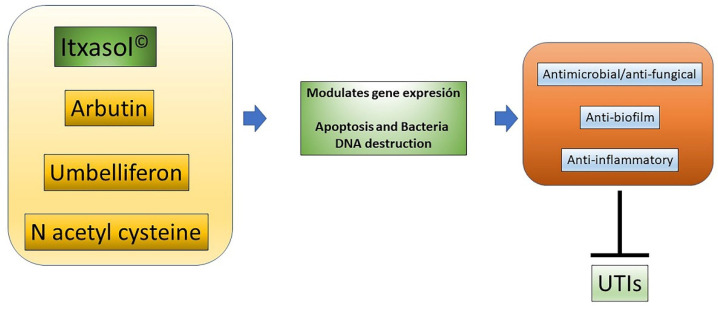
Actions of Itxasol©. The different components of Itxasol© produce different changes in gene expression. To decrease the levels of inflammation in cells and bacteria, they interfere with the structure of DNA or the expression of virulence genes or those involved in the formation of biofilm. In this way, the combined anti-inflammatory and anti-biofilm antimicrobial actions can effectively combat UTIs.

As has been shown, these actions are carried out at least in part through the regulation of bacterial genes related to the expression of virulence factors and the formation of the biofilm, with modification of the expression patterns of genes related to inflammatory processes in different cell types, and with the modification of DNA cells to produce apoptosis (Figure 2). Considering the components of Itxasol©, Umb shows a clear capacity to interfere with the expression of genes related to adhesion and biofilm formation, β-arbutin′s main action is related to the regulation of inflammatory mechanisms, and NAC is able to destroy bacterial DNA and to control the inflammation produced by bacterial LPS. 

At this point, it is necessary to carry out further studies to determine the genetic changes that both bacteria and host cells, including microbiota, have in the presence of the components of Itxasol©, both separately and in conjunction, i.e., resulting from this innovative formulation. As a result of these studies, it will be possible to improve the administration dose and guidelines for this alternative treatment adjuvant and perhaps to master a single administration where there is no need for antibiotics. In this way, it will be possible to improve the treatment of UTIs, reducing the possible adverse effects derived from traditional antibiotics in patients while simultaneously protecting the kidneys.

## Figures and Tables

**Table 1:** Mechanisms of action of the various antibiotics.

Antibiotic	Mechanism of Action	Reference
Nitrofurantoin	Destroys bacterial RNA and DNA	[23]
Fosfomycin	Inhibits Gram positive and negative cell wall synthesis	[23]
Ciprofloxacin	A fluoroquinolone used against Gram negative bacteria that impairs DNA’s bacterial synthesis and inhibits topoisomorases’ actions	[24]
Trimethoprim	Inhibits bacterial folic acid synthesis	[23]
Levofloxacin	Inhibits topoisomerase IV and bacterial gyrase	[25]
Cephalexin	Beta lactam that inhibits cell wall synthesis	[26]
Cefpodoxime	Cephalosporin that inhibits cell wall synthesis	[27]
Ceftibuten	Beta lactam that inhibits cell wall synthesis	[27]
Piperacillin	Beta lactam that inhibits cell wall synthesis	[27]

**Table 2:** Actions of β-arbutin.

Action/Finding	References
Reduces iNOS expression in B2 microglia cells and IL-1β, TNF-α, MCP-1, and IL-6	[53]
Reduces oxidative stress levels in fibroblast and increases apoptosis of tumor cell line	[54]
Increases expression of collagen I	[55]
Decreases osteoclast activity	[56]
No DNA damage in lymphocytes	[57,58]

**Table 4:** Actions of NAC.

Action/Finding	References
Impairs adhesion of bacteria	[68]
Inhibits biofilm formation	[69,70,71]
DNA bacteria and biofilm components are derived from DNA destruction	[72,75]
Inhibits expression of genes related to fibrotic process	[74]
Reduces expression of genes related to apoptosis	[23]

## Data Availability

Not applicable.

## Abbreviations

Umb, umbelliferon; NAC, N-acetyl cysteine; UTIs, urinary tract infections; DNA, deoxyribonucleic acid.
